# Effect of *Lactobacillus acidophilus* & epidermal growth factor on experimentally induced *Clostridium difficile* infection

**Published:** 2011-04

**Authors:** Sukhminderjit Kaur, Chetana Vaishnavi, Kaushal Kishor Prasad, Pallab Ray, Rakesh Kochhar

**Affiliations:** Department of Gastroenterology, Postgraduate Institute of Medical Education & Research, Chandigarh, India; * Department of Medical Microbiology, Postgraduate Institute of Medical Education & Research, Chandigarh, India

**Keywords:** Ampicillin, BALB/c mice, *Clostridium difficile*, EGF, *Lactobacillus acidophilus*, lansoprasole

## Abstract

**Background & objectives:**

*Clostridium difficile*-associated disease (CDAD) remains an important nosocomial ailment. Antimicrobial therapy used for CDAD gives inconsistent results. This experimental study was planned to investigate the beneficial effects of *Lactobacillus acidophilus* and epidermal growth factor (EGF) for CDAD management.

**Methods:**

Among 10 groups of BALB/c mice (6 in each), group 1 served as controls receiving no inoculum. Animals in groups 2-10 received *C. difficile*, those in groups 3, 6 and 9 received *L. acidophilus* and those in groups 4, 7 and 10 received EGF after *C. difficile* inoculation. Animals in groups 5-7 were pre-treated with ampicillin and those in groups 8-10 with lansoprazole prior to *C. difficile*. The animals were killed and investigated for colonisation by *C. difficile* and toxin production, myeloperoxidase (MPO) activity and histopathology.

**Results:**

Colonisation by *C. difficile* was found to be significantly different (*P*<0.001) in the various groups. *C. difficile* toxin titres and MPO activity were significantly lower in animals given *L. acidophilus* and EGF after ampicillin (groups 6 and 7) and lansoprazole (groups 9 and 10). The severity of acute inflammation was also significantly less (*P*<0.05) in caecal and colonic segments of animals in groups 6 and 7 compared to those in group 5. Although the severity of acute inflammation was less in the caecal and colonic segment of animals in groups 9 and 10, the reduction was not significant compared to group 8.

**Interpretation & conclusions:**

Our findings showed that the administration of *L. acidophilus* and EGF reduced the severity of *C. difficile* infection in the experimental animals.

*Clostridium difficile*-associated disease (CDAD) remains an important nosocomial disease resulting in significant morbidity and mortality. In recent years, there has been an upward trend in the incidence of this condition with continuing high rates of recurrent disease^1^. Antimicrobial therapeutic measures *viz*., vancomycin and metronidazole are being used for the treatment of CDAD but with inconsistent results. It has been reported that 80 per cent of the patients with CDAD respond well to the initial treatment of vancomycin or metronidazole. However, the remaining 20 per cent may develop subsequent episodes of CDAD, which may persist over several years, despite repeated antimicrobial treatment^2^. Moreover, neither of the above two antibiotics nor any other antimicrobial regimen is reliably effective in eradicating the *C. difficile* carrier state, probably because the spores of the organism are resistant to their action, unlike the vegetative forms. Severe complications in patients, an increasing frequency of nosocomial outbreaks and cases of recurrent CDAD have necessitated the search for alternative treatments that may be more effective in the management of the disease^3^.

There is an increasing interest in therapeutic approaches employing probiotics to the management of gastrointestinal diseases. Treatment strategies that involve the disruption of intestinal microflora by antibiotics may be particularly effective with probiotics^4^. Probiotics may offer potential effective therapy for CDAD by re-establishing the disrupted microflora, enhancing immune responses and clearing pathogens and their toxins from the host. Several research trials have been done using probiotics for the treatment of CDAD^5^,^6^. However, the lack of definitive evidence regarding efficacy and safety has limited the use of this type of treatment strategy^4^. Similarly epidermal growth factor (EGF), a potent mitogenic peptide that heals gastric ulcers and reduces bacterial colonization^7^ could be investigated as an alternative approach. The literature on the role of EGF in the management of CDAD is scarce. In our recently published study we found that probiotics and EGF reduced the severity of cyclosporine induced CDAD in animals^8^. In the present study BALB/c mice experimentally inoculated with *C. difficile* were investigated to assess the beneficial effects of *L. acidophilus* and EGF for the management of ampicillin and lansoprazole induced CDAD.

## Material & Methods

*Experimental design*: The study was conducted in the Microbiology and Histopathology Divisions of the Department of Superspeciality of Gastroenterology, Postgraduate Institute of Medical Education and Research (PGIMER), Chandigarh, India. The study was approved by the PGIMER Research Ethics Committee and Institutional Animal Ethics Committee. The investigations were conducted from August 2004 to April 2006. Healthy, adult male BALB/c mice, 4-6 wk old and weighing approximately 25 g each were used in the study. The animals (n = 60) included in the experimental study were divided into 10 groups with group 1 serving as controls and receiving no inoculum.

All the animals in groups 2-10 received *C. difficile*. Apart from this, animals belonging to groups 3, 6 and 9 received *L. acidophilus* (LA-5) and those belonging to groups 4, 7 and 10 received EGF for one week after *C. difficile* infection. Animals in groups 5-7 were pre-treated with ampicillin daily for one week and those in groups 8-10 with lansoprazole daily for two weeks prior to *C. difficile* infection. The dosage, period of various inocula given as well as the day of sacrifice of the animals have been given in Table I.

**Table I.** Study design giving the dosage, period of inocula and day of sacrifice of the animals

	Group 1 Control	Group 2 CD	Group 3 CD+PB	Group 4 CD+EGF	Group 5 AB+CD	Group 6 AB+CD+PB	Group 7 AB+CD+EGF	Group 8 PPI + CD	Group 9 PPI+CD+PB	Group 10 PPI+CD+EGF
Ampicillin (66 mg/kg)	_	_	_	_	Day 1-7	Day 1-7	Day 1-7	_	_	_
Lansoprazole (0.5 mg/kg)	_	_	_	_	_	_	_	Day 1-14	Day 1-14	Day 1-14
*C. difficile* (10^8^ cfu/ml)	_	Day 1	Day 1	Day 1	Day 8	Day 8	Day 8	Day 15	Day 15	Day 15
*L. acidophilus* (10^6^ cfu/ml)	_	_	Day 2-7	_	_	Day 9-14	_	_	Day 16-21	_
EGF (100 μg/kg)	_	_	_	Day 2-7	_	_	Day 9-14	_	_	Day 16-21
Sacrifice	Day 8	Day 8	Day 8	Day 8	Day 15	Day 15	Day 15	Day 22	Day 22	Day 22

AB, antibiotic; PPI, proton pump inhibitor; CD, *C. difficile*; PB, probiotic; EGF, epidermal growth factor

*Preparation of various inocula*: A toxigenic strain of *C. difficile* serogroup A (W1194, ATCC 43594) positive for both toxins A and B (kindly provided by Dr M. Delmee, Belgium) was used to prepare the *C. difficile* inoculum (10^8^ cfu/ml) for administration as described earlier^9^. *C. difficile* inoculum was administered orogastrically at a dose of 1 ml/animal. *L. acidophilus* inoculum (10^6^ cfu /ml) was prepared using LactoBacil capsule (Organon India Limited, India). EGF (Sigma, USA) inoculum was prepared in sterile distilled water at a final dose of 100 μg/kg body weight. Ampicillin inoculum was prepared using 500 mg ampicillin capsules (Ranbaxy Laboratories Limited, New Delhi) at a final dose of 66 mg/kg administered daily in two divided doses. Lansoprazole inoculum was prepared using 30 mg lansoprazole capsules (Brown & Burk Pharmaceutical Limited, Tamil Nadu) in sterile distilled water at a final dose of 0.5 mg/kg.

The body weight of the animals was recorded at the start of the experiment (baseline), weekly during the experiment and lastly at the time of sacrifice (final weight). The change in body weight was recorded as the difference of the baseline and the final weight.

All the animals in groups 2-10 were sacrificed one week post *C. difficile* infection using anaesthetic ether via inhalation. The animals in control group were sacrificed along with the animals in group 2 (Table I).

The intestinal tract of each animal was dissected out aseptically using a surgical blade. The colon, caecum and the ileal segments were separated out for further processing. The content from the caecum was immediately washed out with 1 ml sterile saline and this constituted ‘initial dilution’ (ID) for *C. difficile* counts and toxin assays. The colon was washed with sterile physiological saline to clear out the adherent faecal material. Two centimetres of the proximal end was kept in 10 mM proteolysis inhibitor containing ectoin and hydroxyectoin (HiMedia, Mumbai, India) for myeloperoxidase (MPO) activity. The caecum, the ileum and the distal part of the colon were immediately transferred to separate vials containing 10 per cent buffered formalin for histopathological studies.

*C. difficile colonisation*: *C. difficile* counts were assessed in the washed out caecal content by inoculating different dilutions on cefoxitin cycloserine fructose agar and incubating anaerobically at 37°C for 48 h. The colony forming units per ml (cfu/ml) of the original samples were obtained by multiplying the counts obtained with the dilution factor as described earlier^9^.

*C. difficile toxin assay*: *C. difficile* toxins were detected in the caecal content supernatant by latex agglutination assay as described earlier^10^ using antisera for toxins A and B (kindly gifted by Dr M. Warny, USA). All positive samples were subjected to further titration by doubling dilutions. The titre of the toxins (A and B) was recorded as the highest dilution of the supernatant which gave a positive agglutination reaction using *C. difficile* anti-toxin coated latex beads.

*Myeloperoxidase activity*: Myeloperoxidase activity in the colonic tissue was estimated by enzyme linked immunosorbent assay (ELISA) as described by Bradely *et al*^11^ with some modifications. Briefly, colonic tissue (2 cm) was homogenized in 2 ml of 0.5 per cent hexadecyltrimethylammonium bromide (Sigma, USA) buffer (*p*H 6.0) in an ice bath and freezed-thawed for three cycles. It was sonicated on ice for 10 sec and centrifuged at 4472 *g* for 20 min at 4°C. The supernatant was transferred to fresh vial and the protein content estimated^12^. Next, 14 μl of the supernatant was taken in an ELISA plate to which 200 μl of reactive buffer (O-dianisidine and hydrogen peroxide) was added. Incubation was carried out for 15 min at room temperature in dark. Human MPO (Sigma, USA) at a concentration of 0.5 Units/ml was used as a positive control and phosphate buffer (50 mM) as a negative control. The absorbance was read at 450 nm by an ELISA reader (Multiskan, Finland). Each sample was tested in duplicate. The mean MPO value of each sample was expressed as Units/g of protein.

*Histopathological studies*: Formalin fixed specimens of ileum, caecum and colonic segments were processed and stained with haematoxylin and eosin for histopathological evaluation. Particular attention was paid to record the inflammatory changes in the mucosa for surface epithelial injury, change in shape of cells with mucodepletion, presence or absence of erosion/ulcer of surface epithelium, intraepithelial leucocytes (lymphocytes/neutrophils), crypt abscess/cryptitis, lamina propria infiltrate (lymphocytes, neutrophils and eosinophils). The severity of inflammation was graded by a score of 0-3 and represented in the form of mean ± standard error of mean (SEM). Absence of inflammation or no change was recorded as 0; mild, moderate and severe inflammatory changes were recorded as 1, 2 and 3 respectively.

*Statistical analysis*: *C. difficile* colony counts were converted to log_10_values. Mean, median, standard deviation and ranges were calculated. Kruskal-Wallis test was applied to see the significance for parameters like colonisation by *C. difficile*, toxin A and B titres, MPO activity and histopathological changes among the 10 groups. Mann-Whitney U test was applied to compare between two different groups. Analysis of variance test was applied for analyzing significant differences for weight changes. *P*<0.05 was considered significant.

## Results

The animals in control group remained healthy during the experimental period. All the animals in each group survived till the end of the experiment.

There was a significant decrease (*P*<0.05) in body weight of animals receiving *C. difficile* (group 2) compared to group 1. The increase in body weight in the groups receiving probiotic (group 3) and EGF (group 4) was significant (*P*<0.05) compared to group 2, which did not receive either *L. acidophilus* or EGF after *C. difficile* administration. A significant decrease (*P*<0.05) in body weight of animals belonging to group 6 (antibiotic, *C. difficile* and probiotic) was observed compared to controls. Though a decrease in body weight was also observed in group 5 (antibiotic and *C. difficile*) the reduction was insignificant. In contrast to the above two groups, there was an increase in body weight of animals receiving EGF after antibiotic and *C. difficile* administration (group 7). Similarly there was an increase in the body weight similar to the uninoculated controls in all the animals receiving lansoprazole prior to *C. difficile* inoculum (groups 8-10). This increase was more significant (*P*<0.05) in animals receiving probiotic (group 9) compared to those that did not receive either *L. acidophilus* or EGF (group 8) (Fig.).

**Fig.**
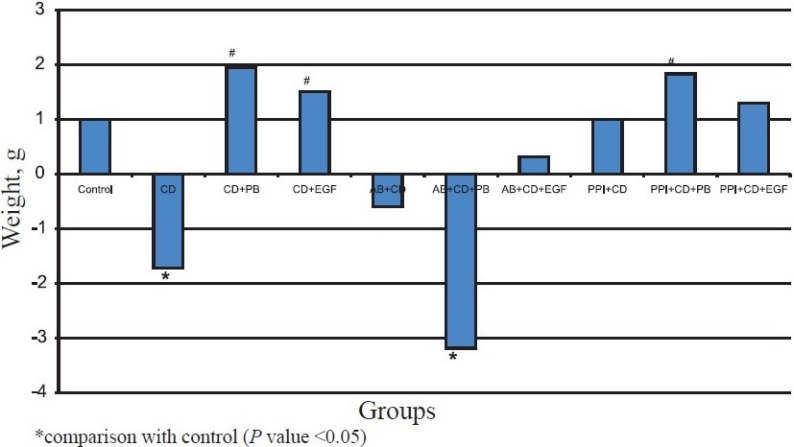
Change in body weight of animals in different groups.

*C. difficile* count differed significantly (*P*<0.001) in all the groups (Table II). During inter-group comparison, *C. difficile* count was significantly reduced in animals receiving either probiotic (*P*=0.003) or EGF (*P*=0.015) in addition to *C. difficile* (groups 3 and 4 respectively) compared to those receiving only *C. difficile* (group 2). *C. difficile* count was significantly lower in animals receiving EGF (*P*=0.004) but did not show significant difference (*P*=0.808) in animals receiving probiotic (group 6) when compared with those not receiving either probiotic or EGF after pre-treatment with antibiotic (group 5). *C. difficile* count was significantly lower in groups 9 (*P*=0.030) and 10 (*P*=0.013) receiving either *L. acidophilus* or EGF in addition to lansoprazole, compared to those not receiving either probiotic or EGF after pre-treatment with PPI (group 8).

**Table II.** Mean cfu/ml, Log_10_ values of *C. difficile* isolated from caecal contents

Group No.	Inocula	Mean	SD	Range	Median
1.	Control	0.0000	0.00000	0.00 - 0.0	0.0000
2.	CD**	4.1833	0.51153	3.40 - 4.90	4.1500
3.	CD+PB*	0.5000	0.54772	0.00 - 1.0	0.5000
4.	CD+EGF**	3.0500	0.77910	2.00 - 4	2.9500
5.	AB+CD**	5.6633	0.56092	5.00 - 6.30	5.8200
6.	AB+CD+PB**	5.6400	0.46217	5.00 - 6.40	5.5500
7.	AB+CD+EGF**	4.1367	0.53850	3.30 - 4.84	4.0400
8.	PPI+CD**	5.2033	0.84687	4.00 - 6.34	5.3000
9.	PPI+CD+PB**	3.7350	1.15765	2.30 - 5.11	3.5000
10.	PPI+CD+EGF**	3.5767	0.95326	2.00 - 4.90	3.6300

CD, *C. difficile*; PB, probiotic; EGF, epidermal growth factor; AB, antibiotic; PPI, proton pump inhibitor

*P* *<0.05

** <0.01 compared to control

*C. difficile* toxins A and B were not detected in animals belonging to groups 1-4 (Table III). However, in animals receiving ampicillin and *C. difficile* (group 5) toxin A was detected in 100 per cent (Positive to 10^3^) and toxin B in 83.3 per cent (undetected to 10^3^) of the animals. With probiotic administration, toxin A was detected in 33.3 per cent (undetected to 10^3^) and toxin B in 50 per cent (undetected to 10^1^) of the animals. In animals receiving EGF (group 7), toxin A was present in 33.3 per cent (undetected to 10^2^) and toxin B in 66.6 per cent (undetected to 10^1^) of the animals. In animals receiving lansoprazole (group 8) toxin A was detected in 83.3 per cent (undetected to 10^2^) and B in 100 per cent (ID to 10^1^) in the caecal content supernatant. In animals receiving probiotic after lansoprazole administration (group 9) toxin A was present in 83.3 per cent and B in 33.3 per cent of them with the titre of both the toxins ranging from undetected to ID. With the administration of EGF (group 10), toxin A was not detected in any of the animals whereas toxin B was present in only 50 per cent of the animals (undetected to positive).

**Table III.** *C.difficile* toxin A and B titres in different groups of animals

	Groups 1-4	Group 5 AB+CD		Group 6 AB+CD+PB		Group 7 AB+CD+EGF		Group 8 PPI + CD		group 9 PPI+CD+PB		group 10 PPI+CD+EGF	
Animal No.	Tox A & B	Tox A	Tox B	Tox A	Tox B	Tox A	Tox B	Tox A	Tox B	Tox A	Tox B	Tox A	Tox B
1	ND	8112	218	ID	ND	256	ID	256	ID	ID	ND	ND	ID
2	ND	ID	ND	ND	ND	ND	ID	128	8	ID	ID	ND	ND
3	ND	8	ID	ND	ID	ND	ID	ND	8	ID	ND	ND	ID
4	ND	ID	ID	8112	16	ND	ND	ID	ID	ID	ID	ND	ID
5	ND	8112	1024	ND	ND	ND	8	128	32	ID	ND	ND	ND
6	ND	ID	512	ND	ID	ID	ND	256	ID	ND	ND	ND	ND

Groups 1-4, Group 1 (Control), Group 2 (CD), Group 3 (CB+PB), & Group 4 (CD+EGF); Tox, toxin; AB, antibiotic; PPI, proton pump inhibitor; CD, *C. difficile*; PB, probiotic; EGF, epidermal growth factor; ND, not detected; ID, initially diluted

Kruskal-Wallis test showed significant difference for toxin A (*P*<0.01) and toxin B (*P*<0.05) among the various groups. Toxin A titres showed significant reduction in animals receiving EGF after antibiotic pre-treatment when compared to group 5. Toxin B titres showed insignificant reduction for toxin B (*P*=0.11), whereas titres for both the toxins reduced significantly (toxin A, *P*<0.01 and toxin B, *P*<0.05) in animals receiving EGF after lansoprazole pre-treatment when compared to those not receiving EGF (group 8).

MPO activity (Table IV) did not show any significant difference in groups 2 to 4 compared to controls. MPO activity was significantly reduced in animals receiving either *L. acidophilus* (*P*<0.01) or EGF (*P*<0.01) when compared to those that did not receive either *L. acidophilus* or EGF (group 5). The MPO activity was also significantly lower in *L. acidophilus* (*P*<0.05) or EGF (*P*<0.05) receiving groups (group 9-10) compared to those not receiving either *L. acidophilus* or EGF (group 8).

**Table IV.** Mean MPO (units/g protein) activity in various groups of animals

Group No.	Inocula	Mean	SD	Range	Median
1.	Control	672.50	335.328	350 - 1123	600.00
2.	CD	1306.33*	292.361	840 - 1747	1322.00
3.	CD+PB	840.33	349.358	538 - 1494	794.50
4.	CD+EGF	926.00	540.573	333 - 1703	839.00
5.	AB+CD	2270.33*	846.725	1316 - 3456	1913.00
6.	AB+CD+PB	1034.33	314.049	419 - 1283	1141.00
7.	AB+CD+EGF	983.67	440.964	479 - 1710	943.50
8.	PPI+CD	1896.83*	861.982	1061 - 3325	1627.00
9.	PPI+CD+PB	974.00	355.947	663 - 1543	812.00
10.	PPI+CD+EGF	1045.17	416.722	327 - 1520	1033.00

CD, *C. difficile*; PB, probiotic; EGF, epidermal growth factor; AB, antibiotic; PPI, proton pump inhibitor

* *P* <0.05 compared to control

No pathological changes were found in the ileal, caecal and colonic segments of animals belonging to groups 1-4. The ileal segments in all the remaining groups showed no pathological alterations. When Kruskal-Wallis test was applied to the inflammatory parameters [mucodepletion (caecum, *P*<0.05; colon, *P*<0.001); neutrophilic infiltrate (caecum, *P*<0.05; colon, *P*<0.001), eosinophilic infiltrate (caecum, *P*<0.05); and cryptitis (caecum, *P*<0.01), a significant difference was found for all the parameters among groups 5-10 except lymphocytic infiltrate. The severity of acute inflammation was significantly lowered (*P*<0.05) in caecal and colonic segments for mucodepletion, neutrophilic infiltrate after administration of *L. acidophilus* compared to those in group 5. Cryptitis was significantly lowered (*P*<0.001) in the caecum of animals receiving probiotic after pre-treatment with antibiotic. The severity of acute inflammation was also significantly lowered (*P*<0.05) in caecal and colonic segments for neutrophilic infiltrate after administration of EGF compared to those in group 5. Although the severity of acute inflammation was lowered in the caecal and colonic segment of animals receiving either *L. acidophilus* or EGF after pre-treatment with lansoprazole, the reduction was not significant compared to those in group 8.

## Discussion

Among the alternative therapies for CDAD, probiotics have been used by many. As it is difficult to do controlled studies in human beings, we have used the murine model for investigating therapeutic approaches like probiotic *L. acidophilus* or EGF in the management of CDAD. The mouse model of *C. difficile* infection has been used successfully for the investigation of histological changes in intestinal mucosa and shifts in intestinal microflora in animal fed with different diets^13^. Wilson *et al*^14^ reported induction of *C. difficile* diarrhoea in cefoxitin-treated conventional BALB/c mice with the morphological findings in the colon similar to changes in the human intestine.

In the present study, the administration of *L. acidophilus* led to a significant increase in body weight in animals in the lansoprazole group. Probiotic administration also resulted in significant reduction of *C. difficile* toxins in animals receiving either ampicillin or lansoprazole. Similar observations were reported by Plummer *et al*^6^ in patients receiving probiotic compared to placebo. Administration of *L. acidophilus* results in its colonisation of the distal end of small intestine and the colon, protecting against *C. difficile* through a ‘barrier effect’ and/or production of antimicrobial components such as bacteriocins. It is also known that lactobacilli are more antagonistic to *C. difficile*, as the former produce hydrogen peroxide and high levels of lactic acid more frequently^15^.

After an inflammatory stimulus, goblet cells, present in the intestine, release mucous. The enhanced presence of goblet cells would insure an improved synthesis of mucous, which improve the intestinal barrier and functioning, and thereby increase host protection against infections^16^. In the present study also mucodepletion was significantly lowered in *L. acidophilus* receiving animals.

Black *et al*^17^ observed the inhibition of *C. difficile* growth *in vitro* by *L. acidophilus* and clinical elimination of relapsing pseudomembranous colitis. Probiotic *L. acidophilus* and *Bifidobacterium bifidum* reduced the incidence of faecal samples positive for *C. difficile* toxins from 7.25 per cent in placebo-control group to 2.9 per cent in probiotic group^6^. However, from their study it was not clear as to which of the two probiotics proved beneficial and whether both benefited in synergy or individually. In the present study, a single probiotic *L. acidophilus* administered showed protective effect against *C. difficile* infection. Gotz *et al*^18^ reported no case of antibiotic associated diarrhoea when a commercial mixture of *L. acidophilus* and *L. bulgaricus* was given to 79 hospitalised patients receiving ampicillin compared to 6 of 43 patients on placebo. However, another study failed to find any protective effect of the probiotic preparation^19^.

We tested the efficacy of EGF in prevention of *C. difficile* colonisation and disease production. Inhibition of bacterial growth by EGF may occur through several ways and need not be due to direct antibacterial effect^20^. Riegler *et al*^21^ described the possible mechanism of the preventive effect of EGF in toxin-induced damage involving binding of EGF to its cell surface receptors and stimulating the tyrosine kinase activity of the receptor. This initiates a signal transduction cascade resulting in biochemical changes within the cell, ultimately leading to DNA synthesis and cell proliferation^22^. High levels of EGF can suppress inflammatory mediators and may stimulate mucosal healing by enhancing the production of other growth factors, including transforming growth factor α and heparin-binding EGF^23^. Besides repair of mucosal surface, EGF plays a protective role in the gastrointestinal tract by accelerated ulcer healing and preventing bacterial invasion as effectively as antibiotic treatment^20^, a property that makes it extremely useful as a therapeutic agent.

Increase in body weight was observed in animals receiving EGF in the present study. Additionally, EGF reduced *C. difficile* count and toxin production significantly regardless of whether an antibiotic or an anti-ulcer drug was given. Buret *et al*^20^ administered EGF to rabbits before oral inoculation with enteropathogenic *E. coli* and demonstrated inhibition of bacterial colonisation, a reduction in microvillus injury and disaccharidase deficiencies.

Mild to moderate inflammation was found in the caecum and colon of animals pre-treated with ampicillin or lansoprazole before *C. difficile* infection in a previous study^9^. Administration of EGF normalised the concentration of inflammatory marker *i.e*. MPO in the colonic segments after *C. difficile* challenge in the present study. This observation is corroborated by significant reduction in the severity of inflammation in the colons of the animals. Less to lesser degree of mucodepletion and neutrophilic infiltration was observed in animals receiving EGF in the drug induced *C. difficile* infections in animals. EGF stimulates epithelial restitution of human colonic mucosa *in vitro*^21^ and promotes enterocyte migration and resurfacing of epithelial discontinuities thereby accelerating the healing of gastric ulcers and colitis^24^.

To conclude, our study confirms the usefulness of probiotic *L. acidophilus* in preventing *C. difficile* colonisation as well as toxin production. Our findings also show the potential benefits of EGF as an alternative strategy in prevention of *C.difficile* infection. However, further clinical trials would be required to use EGF as the therapeutic agent for the management of CDAD.
